# Supplementary material for: The Modernization of Oriental Music Therapy: Five-Element Music Therapy Combined with Artificial Intelligence
Source: Healthcare (Basel). 2024 Feb 5;12(3):411. doi: 10.3390/healthcare12030411 (PMC10855257; doi:10.3390/healthcare12030411)
Supplement: Supplementary file 1 [file healthcare-12-00411-s001.zip › healthcare-2796516-supplementary.pdf]

Supplementary File S1. Notable randomized controlled clinical trials related to five-element music therapy.

1st author (year)	Population	TG	CG	Details of the music therapy	Outcomes and results
Li (2016)[1]	Pregnant women	Conventional care + five-element music therapy (2 weeks)<br>N = 120	Conventional care (2 weeks)<br>N = 120	Every day, the participants listened to Gong tone before nap and Yu tone before bed for 30 minutes each.	1. SAS: TG<CG*<br>2. SDS: TG<CG*<br>3. SF-36<br>1) physical functioning: TG>CG*<br>2) role limitations due to physical problems: TG>CG*<br>3) bodily pain: TG>CG*<br>4) general health perceptions: TG>CG*<br>5) vitality: TG>CG*<br>6) social functioning: TG>CG*<br>7) role limitations due to emotional problems: TG>CG*<br>8) mental health: TG>CG*
Li (2016)[2]	Pregnant women (threatened abortion)	TG1: Conventional care + five-element music therapy (2 weeks)<br>N = 60<br>TG2: Conventional care + medicated diet (2 weeks)<br>N = 60<br>TG3: Conventional care + medicated diet + five-element music therapy (2 weeks)<br>N = 60	Conventional care (2 weeks)<br>N = 60	Every day, the participants listened to Gong tone before nap and Yu tone before bed for 30 minutes each.	1. HAMD: TG1<CG*, TG2<CG*, TG3<CG*, TG3<TG1*, TG3<TG2*, TG1<TG2*<br>2. HAMA: TG1<CG*, TG2<CG*, TG3<CG*, TG3<TG1*, TG3<TG2*, TG1<TG2*
Liao (2013)[3]	Patients with advanced cancer	TG1: Conventional care + five-element music therapy (3 weeks)<br>N = 66<br>TG2: Conventional care + Western music therapy (3 weeks)<br>N = 63	Conventional care (3 weeks)<br>N = 31	The composition of five-element music therapy is performed by the expert. The participants listened to the composed five-element music, 30 minutes per time, 5 days a week.	1. HROLI-R: TG1>TG2+, TG1>CG+<br>2. KPS: TG1>TG2+, TG1>CG+<br>3. Symptom Diary Score: TG1>TG2+, TG1>CG+
Lin (2014)[4]	Patients with Broca's aphasia	Conventional care + five-element music therapy (12 weeks)<br>N = 27	Conventional care (12 weeks)<br>N = 25	The participants listened to Zhi tone, once a day, 30 minutes per time, 5 days a week.	1. CRRCAE<br>1) auditory comprehension: TG>CG*<br>2) repetition: TG>CG*<br>3) speaking: TG>CG*

	after stroke				4) reading aloud: TG>CG*<br>5) reading comprehension: not significant<br>6) copying: TG>CG*<br>7) description: TG>CG*<br>8) dictation: TG>CG*<br>9) calculation: TG>CG*<br>2. CNDS: TGMCG*
Liu (2014)[5]	Elderly with seasonal affective disorder	Conventional care + five-element music therapy (8 weeks)<br>N = 25	Conventional care (8 weeks)<br>N = 25	The participants listened to five-element music. However, the composition method of the five-element music was not described. The music therapy was conducted once a week for 1-2 hours each time.	1. HAMD: TG<CG*<br>2. SDS: TG<CG*

Note. *, p<0.05; +, p<0.01. **Abbreviations**. CG, control group; CNDS, the Clinical Neurologic Deficit Scale; CRRCAE, the Chinese Rehabilitation Research Center Standard Aphasia Examination; HAMA, the Hamilton Anxiety Scale; HAMD, the Hamilton Depressive Scale; HROLI-R, Hospice Quality of Life Index-Revised; KPS, Karnofsky performance score; SAS, the Zung's Self-rating Anxiety Scale; SDS, the Zung's Self-Rating Depression Scale; SF-36, the 36-Item Short Form Health Survey; TG, treatment group.

References

1. Li, Z.-Y.; Yao, L.-L.; Xia, Y.-Q. Effect of five elements music assisted interference on extended duration of pregnancy, maternal emotions and quality of life during the late threatened abortion. *Nursing Practice and Research* **2016**, *13*, 58-60.
2. Li, Z.-Y.; Gan, Y.; J, R. Observation on therapeutic effect of medicated diet combined with five elements music assistant therapy for patients with late threatened abortion. *Chinese Nursing Research* **2016**, *30*, 3868-3871.
3. Liao, J.; Yang, Y.F.; Cohen, L.; Zhao, Y.C.; Xu, Y. Effects of Chinese medicine five-element music on the quality of life for advanced cancer patients: a randomized controlled trial. *Chinese journal of integrative medicine* **2013**, *19*, 736-740, doi:10.1007/s11655-013-1593-5.
4. Lin, R.; Chen, J.; Lin, Q. Effects of the tune of zhi on Broca's aphasia after stroke. *Chinese Journal of Rehabilitation Medicine* **2014**, *29*, 137-140.
5. Liu, X.; Niu, X.; Feng, Q.; Liu, Y. Effects of five-element music therapy on elderly people with seasonal affective disorder in a Chinese nursing home. *Journal of traditional Chinese medicine = Chung i tsa chih ying wen pan* **2014**, *34*, 159-161, doi:10.1016/s0254-6272(14)60071-6.
